# US Medical School Admissions Leaders’ Experiences With Barriers to and Advancements in Diversity, Equity, and Inclusion

**DOI:** 10.1001/jamanetworkopen.2022.54928

**Published:** 2023-02-24

**Authors:** Michelle Ko, Mark C. Henderson, Tonya L. Fancher, Maya R. London, Mark Simon, Rachel R. Hardeman

**Affiliations:** 1 Division of Health Policy and Management, Department of Public Health Sciences, School of Medicine, University of California, Davis; 2 School of Medicine, University of California, Davis; 3 Department of Internal Medicine, University of California, Davis, Davis; 4 Workforce Innovation and Community Engagement, School of Medicine, University of California, Davis; 5 Storywalkers Consulting, Albuquerque, New Mexico; 6 Division of Health Policy and Management, School of Public Health, University of Minnesota, Minneapolis

## Abstract

**Importance:**

Despite decades-long calls for increasing racial and ethnic diversity, the medical profession continues to exclude members of Black or African American, Hispanic or Latinx, and Indigenous groups.

**Objective:**

To describe US medical school admissions leaders’ experiences with barriers to and advances in diversity, equity, and inclusion.

**Design, Setting, and Participants:**

This qualitative study involved key-informant interviews of 39 deans and directors of admission from 37 US allopathic medical schools across the range of student body racial and ethnic composition. Interviews were conducted in person and online from October 16, 2019, to March 27, 2020, and analyzed from October 2019 to March 2021.

**Main Outcomes and Measures:**

Participant experiences with barriers to and advances in diversity, equity, and inclusion.

**Results:**

Among 39 participants from 37 medical schools, admissions experience ranged from 1 to 40 years. Overall, 56.4% of participants identified as women, 10.3% as Asian American, 25.6% as Black or African American, 5.1% as Hispanic or Latinx, and 61.5% as White (participants could report >1 race and/or ethnicity). Participants characterized diversity broadly, with limited attention to racial injustice. Barriers to advancing racial and ethnic diversity included lack of leadership commitment; pressure from faculty and administrators to overemphasize academic scores and school rankings; and political and social influences, such as donors and alumni. Accreditation requirements, holistic review initiatives, and local policy motivated reforms but may also have inadvertently lowered expectations and accountability. Strategies to overcome challenges included narrative change and revision of school leadership structure, admissions goals, practices, and committee membership.

**Conclusions and Relevance:**

In this qualitative study, admissions leaders characterized the ways in which entrenched beliefs, practices, and power structures in medical schools may perpetuate institutional racism, with far-reaching implications for health equity. Participants offered insights on how to remove inequitable structures and implement process changes. Without such action, calls for racial justice will likely remain performative, and racism across health care institutions will continue.

## Key Points

**Question:**

How are US medical school leaders working to advance diversity, equity, and inclusion in admissions given persistent calls to increase diversity in the physician workforce?

**Findings:**

In this qualitative study of 39 deans and directors of admissions from 37 medical schools, participants described embedded processes and structures of institutional racism that hindered efforts to increase diversity in admissions and discussed their strategies to overcome these challenges.

**Meaning:**

The study’s findings suggest that stated diversity commitments will likely remain performative until schools undertake major reforms to remove inequitable incentive structures, funding streams, and faculty and alumni influence as well as internal admissions processes that favor a predominantly White status quo.

## Teaser

This qualitative study uses key-informant interviews to describe US medical school admissions leaders’ experiences with barriers to and advances in diversity, equity, and inclusion in admissions policies and processes.

## Introduction

Despite decades of diversity initiatives in US medical schools, such as affirmative action, Project 3000 by 2000, and diversity standards for accreditation, only affirmative action has brought substantive change, and the consequences of its termination are myriad.^1,2^ The need for physicians from structurally excluded groups, including Black or African American, Hispanic or Latinx, and Indigenous physicians, continues to grow; such physicians broaden access to care, advance cultural humility, and advocate for underresourced communities.^3,4^ From 2021 to 2022, the percentages of both Black or African American and Hispanic or Latinx first-year medical students increased to new highs yet remained substantially lower than their growing proportions in the applicant pool.^5^

Racial inequities in the physician workforce reflect structural and institutional racism in the medical profession.^6^ Structural racism (defined as the social, political, economic, and ideological factors that shape life chances based on race^7^) limits educational opportunities and economic resources for prospective physicians from kindergarten to grade 12 through the undergraduate years, constricting the applicant pool. After medical students matriculate, lack of diversity fosters institutional racism (defined as policies and practices within institutions that produce outcomes that chronically favor a racial group or put a racial group at a disadvantage) that have negative impacts on the learning environment and climate.^8,9,10,11,12,13,14,15,16,17,18,19,20,21^ Lack of racial progress cannot be attributed solely to a small applicant pool or attrition from the profession; medical schools are racialized organizations.^6^

By serving as gatekeepers to the profession, admissions offices can perpetuate or counter racism within the medical field. Admissions committee members have cited the Medical College Admissions Test (MCAT), lack of faculty diversity, and inadequate institutional resources and commitment as barriers to increasing diversity.^22,23,24,25^ Given long-standing awareness of such issues and repeated national campaigns, it remains unclear whether admissions leaders have pursued reforms and what happened if they did. Racialized organization theory posits that processes that appear impartial, such as admissions policies and practices, may passively sustain institutional racism.^26^ In this qualitative study, we aimed to describe US medical school admissions leaders’ experiences with barriers to and advances in diversity, equity, and inclusion by investigating their definitions of diversity and inclusion, exploring their strategies and challenges, and highlighting the ways in which racism operates within medical schools.

## Methods

### Qualitative Approach and Research Paradigm

In this cross-sectional qualitative study, a phenomenological approach was used to understand admissions leaders’ experiences, perspectives, and motivations with respect to diversity and inclusion. The study protocol was approved by the institutional review board of the University of California, Davis. During recruitment and before each interview, we informed participants of the study objectives and obtained verbal consent to record, transcribe, and publish quotations without identifying information. This study followed the Standards for Reporting Qualitative Research (SRQR) reporting guideline.^27^

### Researcher Characteristics and Reflexivity

Team members identify as Asian American (M.K.), Black (R.R.H.), White (M.C.H., M.R.L., and M.S.), and multiracial (T.L.F.). Study design, analysis, and interpretation were informed by our differential experiences by race, gender, and power within academic institutions and our collective experiences serving on admissions committees, overseeing the admissions process, and facilitating discussions on diversity, equity, and inclusion.

### Context and Sampling Strategy

We recruited deans and directors of admissions at US allopathic schools of medicine from October 16, 2019, to March 27, 2020, using personal networks, advertisement at the Association of American Medical Colleges (AAMC) Learn Serve Lead 2019 meeting, and direct email correspondence. Information from interviews was analyzed from October 2019 to March 2021. We conducted purposive sampling to obtain a range of perspectives across school racial and ethnic composition. Using 2018 to 2019 data,^28^ we ordered schools by percentage of students who have been historically and structurally excluded from the medical profession (defined as students who identified as American Indian or Alaska Native,^29^ Black or African American, Hispanic or Latinx, and/or Native Hawaiian or Pacific Islander), determined quartiles of student diversity (with quartile 1 comprising medical schools with the greatest diversity and quartile 4 comprising medical schools with the least diversity), and targeted recruitment for representation from each quartile.

We used the term *structurally excluded* to signify that certain racial and ethnic minority groups have been (and continue to be) excluded from the profession as a reflection of active rather than passive processes. Notwithstanding the lack of attention to Indigenous populations, we considered American Indian or Alaska Native and Native Hawaiian or Pacific Islander populations to be groups that have experienced substantial historical and structural exclusion. School-specific definitions of the term *underrepresented in medicine* (URiM; based on groups underrepresented relative to the population) and corresponding demographic characteristics were not publicly available. Therefore, in our reported findings, we used URiM when it was referenced by study participants.

To preserve confidentiality, we aggregated participant race and ethnicity into 4 major categories (Asian American, Black or African American, Hispanic or Latinx, and White); none of the participants identified as American Indian or Alaska Native, Native Hawaiian or Pacific Islander, or other Indigenous groups. For those identifying as multiple races and ethnicities, all races and ethnicities were counted; therefore, the number of participants in racial and ethnic categories exceeded the total number of participants in the study.

### Data Collection and Analysis

We conducted in-person and online interviews (with M.S. acting as the primary interviewer and M.K. and M.C.H. acting as secondary interviewers); the interview guide is available in the eAppendix in Supplement 1. Interviews ranged from 30 to 90 minutes and were audio recorded, transcribed, cleaned of additional identifying information, and stored using an alphanumeric study identification number. We imported and analyzed transcripts using Dedoose software, version 7.0.23 (SocioCultural Research Consultants).^30^ Participants completed a survey that included self-identified gender, race and ethnicity, years of experience in admissions, and a personal background prompt (“Please indicate any terms that describe you: socioeconomically disadvantaged during childhood/adolescence, first generation to college, have a disability”).

We used a combined deductive and inductive approach. Two reviewers (M.K. and R.R.H.) conducted 2 rounds of independent review, comparison, and resolution of transcripts to develop initial and final codes. One reviewer (M.K.) then conducted a final coding of all transcripts to produce categories, which were reviewed by the full team.

To enhance trustworthiness, we created an audit trail, including detailed memos on coding and analysis, and we used investigator triangulation across team members’ experiences to determine preliminary and iterated themes. Investigators who coded the transcripts conferred with other team members with specific admissions leadership experience to confirm and/or add context.

## Results

Thirty-nine deans and directors of admissions from 37 institutions, with 8 to 12 leaders from each medical school diversity quartile, participated in the interviews (Table 1). Overall, 56.4% of participants identified as women, 10.3% as Asian American, 25.6% as Black or African American, 5.1% as Hispanic or Latinx, and 61.5% as White (participants could report >1 race and/or ethnicity). Admissions experience ranged from 1 to 40 years. In total, 28.2% of participants identified as socioeconomically disadvantaged in childhood, and 30.8% identified as first-generation college students.

**Table 1.** Participant Characteristics: US Allopathic Medical School Admissions Deans and Directors

Characteristic	Participants, No. (%) (N = 39)
Years of experience in admissions	
0-5	3 (7.7)
6-10	9 (23.1)
11-20	16 (41.0)
≥21	11 (28.2)
Gender^a^	
Woman	22 (56.4)
Man	17 (43.6)
Race and ethnicity^b^	
Asian American	4 (10.3)
Black or African American	10 (25.6)
Hispanic or Latinx	2 (5.1)
White	24 (61.5)
Rural origin	
Yes	5 (12.8)
No	34 (87.2)
Socioeconomically disadvantaged background^c^	
Yes	11 (28.2)
No	28 (71.8)
First generation to attend college^c^	
Yes	12 (30.8)
No	27 (69.2)
Medical school diversity quartile^d^	
1	12 (30.8)
2	8 (20.5)
3	9 (23.1)
4	9 (23.1)
HBCU	1 (2.6)

Abbreviation: HBCU, historically Black college or university.

^a^ Self-identified by participants in an open-ended response item. No participants identified as a gender other than woman or man.

^b^ To preserve confidentiality, we aggregated race and ethnicity into 4 major categories. For those identifying as multiple racial and ethnic identities, all races and ethnicities were counted; thus, the number of participants in racial and ethnic categories exceeded the total number of participants in the study. No participants identified as American Indian or Alaska Native, Native Hawaiian or Pacific Islander, or other Indigenous groups.

^c^ Participants responded to the following item: “Please indicate any terms that describe you: socioeconomically disadvantaged during childhood/adolescence, first generation to college, have a disability.” No participants reported having a disability.

^d^ Participants’ institutions were ranked in quartiles according to the percentage of enrolled medical students who identified as one or more of the following races and ethnicities in 2019: American Indian or Alaska Native, Black or African American, Hispanic or Latinx, and/or Native Hawaiian or Pacific Islander. Quartile 1 consisted of medical schools with the greatest student diversity, and quartile 4 consisted of medical schools with the least student diversity. Given their unique history and mission, HBCUs were identified separately.

### Meanings and Discussion of Diversity

All participants initially defined diversity by listing race and ethnicity but immediately qualified their answers by asserting that diversity captures “much more,” such as life experiences, perspectives, sexual orientation, gender, rurality, military service, college athletic activities, and religion (Table 2). While participants said that diversity was discussed “everywhere” in their institutions, most felt that only student admissions offices had made substantive diversity gains. Two participants expressed concern that the shift to a generalized definition of diversity had supplanted attention to unresolved racial inequities.

**Table 2.** Medical School Admissions Leaders’ Constructed Meanings and Perceptions of Diversity

Theme	Supporting quotes
Diversity encompasses more than race and ethnicity	We tell our students, every one of you is diverse.
	Diversity in America these days usually means Black, Hispanic and White. In medical education, at our institution, it means diversity of human beings.
Diversity is discussed everywhere but not effective	It always becomes really complicated and I’m still trying to figure out how we know when we get there, but we just know we’re not there yet.
Diversity displaces racial justice	Race and ethnicity get pushed to the side when you start to look at all those things.
	There is a risk of claiming diversity without solving those most important historical issues of racial and ethnic discrimination.

### Challenges to Increasing Diversity in Medical School Admissions

Irrespective of how participants defined diversity, they described challenges specific to racial and ethnic diversity efforts (Table 3). First, participants cited senior leadership difficulties, which included (1) frequent turnover, leading to loss of leaders with a commitment to diversity, and a reluctance among interim leaders to enact changes; (2) shifting priorities, in which initial commitments were later abandoned for other causes; and (3) performative attention without supporting policies or resources.

**Table 3.** Challenges to Increasing Racial and Ethnic Diversity Within Medical School Admissions

Theme and subtheme	Supporting quotes
**Challenges faced by all schools**	
Leadership inadequacies	
Leadership turnover	If you’re an interim dean, you’re just trying to maintain status quo…less likely to make waves, less likely to make bold steps in times when they're necessary.
	The dean who was here was promoting a lot of great ideas and initiatives…it has eroded in every iteration of that dean since.
Lack of commitment	The hierarchy doesn’t listen….Year in, year out, it’s always something else given priority. Research, building new buildings.
Performative display	That’s been historically not just the school of medicine, but the entire institution, “Let’s look good on paper.”
	Conversation among-within admissions and outside admissions, is that diversity…is a check box, that it's just counting bodies and not really supporting folks.
Undue emphasis on academic measurements	
Prioritized over other applicant qualities	It's not about diversity anymore—this scrutiny of MCAT even beyond the point where we have already considered it.
Perceived predictors of future success	We’re all risk-averse and we like to rely on these numbers that we think will determine success, but it’s not the whole picture.
Importance for school rankings	The thing we dare not speak out is, how do you accomplish taking students who don’t do as well on standardized exams, and balance that with the *US News & World Report* and the reputation of the school?
Convenience	It’s become increasingly difficult, when the number of people applying, is so huge, not to be seduced by grades and MCAT.
Social and political connections	
Formal processes	[Awarded] bonus points if you knew somebody, or your father was an alumni of the institution, or somebody was a donor, and that moved [you] up on the alternate of the wait list.
Informal pressure	After our white coat ceremony, [my dean] received a lot of questions [from alumni and faculty], “Whatever happened to the six-foot-two blonde, White boys we used to have in our medical school, where did they all go?”
	The development office gets lots of inquiry for particular faculty, since they have produced so much for the institution. Imagine how difficult it would be to say, no [their relative] is not coming.
**Challenges specific to schools with lower levels of student diversity**	
Competition for URiM applicants^a^	
Small URiM applicant pool	The number is so small in this country that we are fighting each other.
Role of scholarship funding	[It’s] a situation where you are catching up money to give to minorities, to almost bribe them to come to your institutions.
	There used to be a show on, many years ago, called *Let's Make a Deal*, and I feel like I'm making a deal. I don't want to buy a student.
**Challenges specific to schools with higher levels of student diversity**	
Lack of inclusive environments^b^	
Inadequate faculty and resident diversity	We have a number of upper-echelon committees and a lot of times I’m the only person of color. It’s almost like colonialism.
	The students are like, “You brought me here for what? Where’s the faculty, where’s the leadership?”
Implicit bias against URiM students	No matter how I blind [academic measurements], interviewers come back and ask, “How did you do on the MCAT”.…They tend to ask Black women and men that question, but they don’t ask White people that question.
	The response [faculty] give will be condescending, almost to the point of being demeaning. Our students recognize it and they stop going to office hours. Yet the professor will say, “So, I have these office hours and they don't come.” They don't come because you treated them like crap.
Overt hostility from medical school community	When students who weren’t doing well, [teaching faculty] were blaming admissions [for accepting more URiM applicants].… Students felt very marginalized and attacked.
	[My predecessor] faced a lot of hostility from the majority students. Threats to get [them] fired.
Discouragement of URiM applicants	Now [applicants] are talking to 3rd and 4th years, who have had a less than ideal experience. Applicants are less likely to matriculate unless we give them some huge bonus to get here.
Burnout among admissions leaders	I thought I was going to have a stroke my first year, because they were doing things that…just about killed me. It got me on high blood pressure medications, I kid you not.
	It’s a period of pushback, voicing their opinions, then they get to the point where I just want to finish and leave, and I never want to come back.

Abbreviations: MCAT, Medical College Admissions Test; URiM, underrepresented in medicine.

^a^ More dominant theme among participants from schools in the bottom half of URiM representation.

^b^ More dominant theme among participants from schools in the top half of URiM representation.

Second, most participants reported that undue emphasis on academic scores, particularly MCAT scores, hampered their work. All participants acknowledged that persistent structural disadvantages (rather than differences in ability) contributed to lower mean MCAT scores among those labeled as URiM. Justifications for overweighting MCAT scores included (1) predictive value for medical school success, namely licensure examinations; (2) convenience for screening a large volume of applications; and (3) the school’s *US News & World Report* rankings. One participant explained that while scores constitute only a fraction of the *US News & World Report* ranking calculation, matriculant scores are one of the only domains by which schools can exercise direct influence on their ranking. Consequently, senior leadership regularly pressured admissions officers to prioritize MCAT scores.

Third, participants listed the ways in which applicants with social and political connections may have advantages in the process, including through offering additional reviews, “keeping an eye out” for a given application, automatically granting interviews, and maintaining a database of preferred applicants. Many participants emphasized that these connections were not considered in final acceptance decisions. However, a few participants acknowledged that these intermediate steps favored applicants with racial and socioeconomic advantages.

When comparing responses by institutional diversity, we found divergent themes (Table 3). Participants from schools with lower diversity expressed frustration with intense competition for a small pool of applicants and an increasing need for scholarship funds. One participant reported that, in the past, elite schools colluded to negotiate scholarships and offer acceptances to URiM applicants. The participant felt that when the AAMC stopped sharing multiple acceptance information with schools in 2019, competition across all schools inadvertently increased because they could no longer rely on hidden agreements to secure the students they wanted.

In contrast, participants from schools with higher diversity reported the lack of inclusive environments as their major challenge (Table 3), citing inadequate faculty and resident diversity, instructors’ implicit biases, and overt hostility from faculty, alumni, and other students. Participants expressed concern that negative environments damaged their school’s reputation among applicants, which then detracted from their recruitment efforts. A few participants expressed experiencing substantial burnout due to increasing student diversity within an unsupportive environment, and some participants had predecessors who had left the institution for these reasons.

### External Influences on Diversity Efforts

Participants described the ways in which external policy and organizational factors affected their work (Table 4). Many reported that Liaison Committee on Medical Education (LCME) accreditation standards motivated their institutions to support diversity efforts in admissions. However, some cautioned that LCME standards, which are self-defined, led to a down-leveling effect such that schools pursued lowered diversity objectives that could be achieved without major reforms. Many participants referred to AAMC initiatives on holistic review. Some reported that such efforts had helped them garner institutional support for reforms, while others dismissed holistic review as a rebranded diversity effort. Some characterized holistic review as a performative process that could unintentionally counteract diversity efforts (Table 4). Another participant explained that when admissions ratings systems reward applicants for cumulative hours or number of experiences (without attention to how those experiences should be valued), applicants with socioeconomic advantages benefit because they have more time and resources for such extracurricular opportunities. Participants also stated that the legal and policy environment overshadowed all racial and ethnic diversity work; several participants expressed concerns about legal liability in the use of race and ethnicity in admissions, irrespective of whether their institution was subject to such a ban.

**Table 4.** External Influences on Medical School Admissions’ Diversity Efforts

Actor	Influence	Supporting examples
LCME	Institutional motivation	Once you put anything into an LCME standard, you have made a statement—that this is important to your survival.
	Down-leveling	The LCME is looking at total workforce diversity. That’s where the enterprise felt like they were going to fall down, and they didn’t want to put themselves in that position [by including American Indian representation in diversity goals].
AAMC	Holistic review initiatives as support for diversity efforts	When you don’t focus entirely on the metrics you can increase diversity of your class and AAMC has showed us that that that is the case.
	Holistic review as a rebranding of diversity	[Leadership says] we’ll introduce holistic admissions to increase diversity. I think that’s really a misuse of holistic admissions.
	Encouragement of performative rather than substantive change	My previous institution had good lip service. They love to say that they want to do a holistic process, but all they did was look at numbers. They would kill people who had bad numbers, despite having everything else look good.
State and local policy environment		
Barriers	State bans on race and ethnicity considerations	[Race consideration] was taken away from us.…It’s really hard to look at your numbers after a cycle and realize that you’ve taken a step backward.
	In-state admissions requirements	I’m stuck between a rock and a hard place, because I need to accept a fixed percentage from within the state [but] I’m in a demographically challenging state.
	State mandates for academic thresholds	[URiM] applicants who take the MCAT—their average is [only a few points higher than the state requirement].
Supports	State support for underserved communities	We have one geographical mandate, which is a part of our state which is typically a rural, underserved, and large populations of minority patients.

Abbreviations: AAMC, Association of American Medical Colleges; LCME, Liaison Committee on Medical Education; MCAT, Medical College Admissions Test; URiM, underrepresented in medicine.

### Strategies to Advance Diversity in Medical School Admissions

Participants articulated several strategies to advance diversity in medical schools. These strategies included changing the story, the process, the people, and the organization (Table 5; a detailed list of strategies is available in the eTable in Supplement 1).

**Table 5.** Types of Strategies Used by Admissions Leaders to Increase Diversity

Theme	Strategy
Change the story	Reframe responsibility of admissions
	Incorporate racial justice into institutional mission
	Tailor narratives for messenger and context
Change the process	Institute mission-specific measurements, rubrics, and processes
	Eliminate preferences for alumni, faculty, and development connections
	Reform use of academic measurements to reflect relative importance to mission
	Use a continuous quality improvement approach
Change the people	Hire URiM staff
	Recruit URiM committee members and provide compensation
	Invest in URiM outreach and recruitment
	Build pathway programs with local and regional minority-serving institutions
Change the organization	Institute diversity accountability across all units
	Elevate admissions leadership in organizational structure
	Increase student representation and engagement across units
	Provide student support services specific to URiM students’ needs
	Require health equity training in curriculum
	Commit institutional resources and authority to offices of diversity, equity, and inclusion

Abbreviation: URiM, underrepresented in medicine.

#### Change the Story

Several participants used narrative change to support their work, with 2 main themes emerging (Table 5). First, they reframed the purpose of admissions, which is summarized as follows: the responsibility of admissions is to select students for their potential as future physicians, not for their guaranteed success in school. The responsibility for academic success is shared with student support services and medical education. Participants described this as a counter-narrative to prevailing norms that prioritize selecting students for their predicted likelihood of success on standardized tests, irrespective of the schools’ curriculum or educational milieu.

Second, they advocated for changing the institutional mission to guide admissions priorities, which is summarized as follows: the institutional mission is to serve historically and currently underserved racial and ethnic minority populations. Defining racial and ethnic minoritized populations allowed leaders to set priorities in academic measurements, educate committee members on their use, and increase transparency in the admissions process.

Receptivity to narratives varied by the messenger’s race and ethnicity. White participants frequently cited the benefit of using quantitative data to dispel myths about academic scores and to reinforce narratives of success among URiM students, while non-White participants encountered resistance when articulating similar narratives to their institutional leadership, even with supporting data.

#### Change the Process

Participants instituted an array of process changes (Table 5; eTable in Supplement 1). They developed mission-based measurements throughout the process, modified or eliminated initial screening by academic measurements, and capitalized on recent undergraduate admissions scandals to eliminate advantages for those with social and political connections. To reinforce change, several participants adopted a continuous quality improvement approach, collecting and analyzing data and providing committee feedback throughout the admissions season.

#### Change the People

Most participants reported that admissions committee members received implicit bias training, which varied from a single online module to multiday in-person workshops. They expressed mixed opinions on the efficacy of training, favoring other strategies, such as reconstituting committees and staff for greater diversity, establishing a shared commitment to reform, and creating an environment that supports open discussion. Not all participants had the ability to select their committee members without faculty elections and/or approval, and they lacked support for proposed changes due to inadequate diversity among faculty.

#### Change the Organization

Participants emphasized that all units must be engaged in diversity work rather than admissions operating in a silo. Those who expressed the greatest satisfaction worked in partnership with or held roles in offices of student affairs, education, or diversity, equity, and inclusion. Establishing collective responsibility helped them manage relationships with senior leadership, particularly among nonphysician and non-White participants. Those who could report directly to the dean reported greater protection from opponents, reinforced credibility, greater prioritization of their work, and increased leadership accountability.

Many participants felt that including students in the admissions process proved valuable because senior leadership was more responsive to student concerns. Participants from schools with a higher level of diversity stated that student input had prompted school changes in support services and curricular reforms. Furthermore, they emphasized that such changes are critical for fostering an inclusive educational environment, which not only benefits racial and ethnic minority students but also impacts the ability of admissions officers to recruit a more diverse applicant pool in the next cycle.

## Discussion

In this qualitative study of 39 deans and directors of US medical school admissions, we found that admissions leaders held various interpretations of diversity, continued to face challenges to increasing racial and ethnic diversity, and engaged in multiple strategies to overcome these barriers.

### Meanings and Uses of Diversity

Our findings are consistent with research reporting the ways in which institutions of higher education shifted from defining diversity as specific to racial and ethnic inequities to a safer and more ambiguous concept of diverse experiences and perspectives that are unrelated power structures.^31^ James Thomas,^32^ an educational sociology researcher, described this process as condensation, which undermines reforms by diverting attention from the distribution of power and privileges based on race. In contrast to earlier research, none of the participants characterized *diversity* as a necessary term to comply with legal prohibitions against the use of race and ethnicity in admissions considerations.^33^ All participants proceeded to discuss race and ethnicity initiatives, suggesting that definitions of diversity arise in part from their institutional structures.

Ambiguous expectations may explain why participants felt that only admissions offices had made substantive progress within their schools. Thomas^32^ described this process as flattening, in which diversity is discussed and promoted everywhere but practiced nowhere. As Ahmed,^34^ a feminist scholar, has explained, when academic units are not held accountable, merely discussing diversity may become conflated with doing real diversity work. Participants in our study had greater racial and ethnic diversity than typical populations of physicians and deans,^35^ potentially explaining their greater attention to these issues and higher expectations of their institutions.

We found that holistic review may perform rhetorical work that is similar to the term *diversity*. While holistic review initiatives have been associated with increases in student racial and ethnic diversity,^36^ the concept does not explicitly call attention to racialized privileges. Our findings suggest that admissions leaders aiming to reform their processes may inadvertently replicate a system of unearned advantages. Flattening and condensation regarding holistic review^37^ may explain why some participants found holistic review to be an effective diversity strategy, while others did not. Holistic review is an important guiding principle but should not be conflated with specific justice-based criteria, continuous internal evaluation, and institutional accountability.

### Persistent Challenges

According to Thomas^32^ and Garces and Cogburn,^38^ when diversity does not explicitly address racial and ethnic inequity, schools can maintain White-centered frameworks and norms in their admissions processes. Participants described common features of unsuccessful diversity initiatives, including inconsistent leadership, inadequate resources, and performative quick fixes.^32,34^ They described goals and strategies, but our findings suggest medical schools have not remediated the overarching structural conditions.^6^

Participants explained why their admissions processes overweighted academic scores, even when they personally disagreed with the practice. Previous studies^31,39,40,41,42^ have noted cultural resistance to change in medical schools, particularly the ways in which administrators and faculty frame diversity as oppositional to merit. These studies^31,39,40,41,42^ did not examine racialization of contexts.^43,44^ Critical race theorists studying institutions of higher education have explained that the perceived conflict between merit and diversity is rooted in underlying biases about the capabilities of racially minoritized individuals.^45^ These biases prevent faculty and administrators from considering diversity as an integral value.^46,47^ Admissions leaders have less opportunity to enact reforms if they must also fight entrenched institutional White supremacy.

Our findings are consistent with selective inclusion, a term coined by Berrey^33^ to describe the process by which institutions admit a small number of individuals who belong to minoritized racial and ethnic groups in the name of diversity but maintain traditional admissions policies and practices to preserve institutional reputation. National rankings systems convert academic scores to prestige, which increases fundraising capability.^48^ School reputation and financial incentives reward practices such as MCAT screening and maximizing scores, even when these practices have been associated with lower racial and ethnic diversity in the student population.^49,50^ Participants explicitly described the ways in which deans, development offices, faculty, and alumni all intervene in admissions practices for these reasons. With increasing application volume, using the MCAT for screening provides administrative efficiency while potentially eliminating qualified applicants. This practice is consistent with an organizational culture in which admissions leaders are expected to adapt to structures rather than modify them.^26^

Conferring advantages to applicants with social connections reinforces selective inclusion by rewarding the prevailing racialized status hierarchy. Some participants engaged in practices that, due to structural racism, disadvantaged American Indian or Alaska Native, Black or African American, Hispanic or Latinx, and Native Hawaiian or Pacific Islander applicants. Those who attempted to remove social privileges encountered resistance from deans, faculty, and alumni, illustrating another way in which organizational culture supports institutional racism.^26^ Perceived threats to majority interests may lead to retrenchment and collusion behaviors as well as hostility.^38^ This reaction may explain why 1 participant felt that admissions leaders should advocate for removing social privileges to protect schools from legal liability rather than arguing that such changes are needed to improve diversity. The prospect of litigation is also a concern among majority stakeholders.

Participants from schools with lower racial and ethnic diversity operated from the perspective of scarcity (ie, too few qualified applicants), whereas those from more diverse schools focused on increasing the applicant pool. Our study was not designed to ascertain causal effects, but findings suggest a need for greater investment in pathway programs and recruitment and more inclusive medical school environments. We noted that schools that made progress in admissions practices in 1 year risked losing these gains if school climate and curriculum were not also addressed.

The persistence of admissions challenges highlighted how pervasive organizational factors could be in weakening initiatives to advance racial equity. Our findings underscore the ways in which admissions policy and practice may reproduce and be shaped by institutional racism.

### Internal Strategies to Increase Diversity

Ray^26^ described the importance of both internal and external opportunities to dismantle racism within organizations. A few participants conducted a detailed audit of their internal processes, which allowed them to interrogate and upend traditional practices. Participants’ internal strategies included developing a mission-based review process and diversifying committee membership.^41,48,51,52^ Notably, our study illustrated the ways in which participants tried to implement change. Several participants referenced the importance of narrative change, a key element of critical race theory known as counter-storytelling.^53^ Counter-storytelling reframed the purpose of admissions, shifting the focus to awarding the assets offered by structurally excluded applicants rather than fighting against deficit narratives. However, even this strategy was contingent on the racialized power structures that determine the credibility of the narrator. This issue reflects another avenue by which academic medical programs undervalue and underrecognize the contributions of racial and ethnic minority leaders.

Our findings reinforce the long-standing assertion that schools must pursue multiple coordinated strategies by changing the story, people, process, and organization together. Beyond diversity goals, admissions leaders need committee member representation, standardized evaluation measures, and procedures that have clear capability to end racial inequities in the medical profession.^47^ Collaborating with other units, particularly medical education (eg, curriculum), may overcome institutional silos that continually burden diversity work, preventing coordination and reducing collective responsibility.^32,34^ Participants felt most effective when they had supportive colleagues, authority to reform processes, and protection from other actors by senior leadership. Admissions leaders have no shortage of methods for advancing diversity (Table 5; eTable in Supplement 1), but pursuing these methods as solo efforts can lead them back to the same barriers encountered by their predecessors.

### External Opportunities for Change

Participants in our study described using a few external strategies. They reported that the LCME accreditation standards could stimulate accountability and motivate medical school leadership. The AAMC holistic review tools, which are designed to specifically address racism,^54^ have lent credibility to admissions leaders advocating for institutional reform. To sustain long-term change, medical schools should extricate themselves from larger systems that conflict with their missions and should instead work with policy makers and funders to secure support for initiatives that prioritize training physicians from, and for, underresourced communities.

### Limitations

This study has several limitations inherent to a cross-sectional key-informant qualitative study. First, we spoke with admissions leaders, not individual committee members. In a previous study of holistic review,^51^ committee members reported confusion about the purpose of the initiative, increasing their resistance to proposed AAMC reforms. Second, our study should not be considered representative of US medical schools; consistent with qualitative paradigms, we aimed for representation of perspectives rather than populations. Given the differing objectives of osteopathic medical schools, we did not sample from these institutions. However, because the number of osteopathic physicians has grown rapidly in the past decade, investigation of their admissions processes is needed. Third, we conducted our study before the COVID-19 pandemic and the renewed attention to racial justice after the murder of George Floyd; whether these events prompt sustained changes in admissions policy and practice remains to be seen.^6^ For the 2021 to 2022 academic year, Black or African American and Hispanic or Latinx enrollment increased but at a rate 50% lower than their increase in the applicant pool.^5^ This discrepancy may reflect the fact that the structural dimensions of medical schools, including rankings, funding imperatives, and lack of faculty diversity, remain largely unchanged. Our study period does not cover recent cases heard by the US Supreme Court, in which plaintiffs have argued that considerations of race and ethnicity in admissions discriminate against Asian American and White applicants.^55^ The implications of these legal cases for participant perspectives are unclear; previous research^56^ found that state bans on affirmative action produced some secondary consequences for private institutions and states without bans. Fourth, this study examined admissions policies and practices with respect to institutional racism. Academic medical programs also need to address other forms of institutional oppression, including ableism and heterosexism and their intersectionality, which can all be weakened under the umbrella of diversity. While beyond the scope of our study, these issues require additional investigation.

## Conclusions

This qualitative study found that medical school admissions leaders encountered multiple constraints within the current organizational and power structures of academic medical programs. Comprehensive institutional- and process-level reforms are needed to dismantle admissions systems that perpetuate institutional racism. Increasing the racial and ethnic diversity of the physician workforce is foundational for achieving health equity. Academic medical leadership should pursue both internal and external opportunities, as there is still much work to be done.

## Notes

Supplement 1. **eAppendix.** Interview Guide **eTable.** Detailed Examples of Strategies Used by Admissions Leaders to Increase Diversity and Inclusion in US Medical Schools

Supplement 2.
Data Sharing Statement
