# Supplementary material for: US Medical School Admissions Leaders’ Experiences With Barriers to and Advancements in Diversity, Equity, and Inclusion
Source: JAMA Netw Open. 2023 Feb 24;6(2):e2254928. doi: 10.1001/jamanetworkopen.2022.54928 (PMC9958522; doi:10.1001/jamanetworkopen.2022.54928)
Supplement: Supplement 1. — eAppendix. Interview Guide eTable. Detailed Examples of Strategies Used by Admissions Leaders to Increase Diversity and Inclusion in US Medical Schools [file jamanetwopen-e2254928-s001.pdf]

Supplemental Online Content

Ko M, Henderson MC, Fancher TL, London MR, Simon M, Hardeman RR. US medical school admissions leaders’ experiences with barriers to and advancements in diversity, equity, and inclusion. *JAMA Netw Open*. 2023;6(2):e2254928. doi:10.1001/jamanetworkopen.2022.54928

eAppendix. Interview Guide

eTable. Detailed Examples of Strategies Used by Admissions Leaders to Increase Diversity and Inclusion in US Medical Schools

This supplemental material has been provided by the authors to give readers additional information about their work.

© 2023 Ko M et al. *JAMA Network Open*.

eAppendix. Interview Guide

Thank you again for taking the time to participate in our study. As we discussed, our aims are to understand current approaches to admissions across U.S. medical schools, how they have evolved over time, and the internal processes – for example, how are admissions policies developed and who is involved.

Please note that this interview will be recorded and transcribed. Your participation is voluntary: you can choose not to answer a question or you can withdraw at any time. We take the confidentiality of your responses very seriously. Your identifying information and that of your school are not attached to this interview. You are not expected to state names, including your own and those of colleagues, your institution, and your location. If you do, be assured we will delete any identifying information from the transcript.

1. **Tell me about your role related to medical school admissions.**

2. **How, if at all, has your school's approach to admissions changed over the past ten years?** (*If the participant has been at multiple institutions, ask to describe their perspective of changes over time across their current and prior institutions*)

Probes:

- *Why were those changes instituted?*
- *Who initiated them?*
- *What were challenges in implementing change?*
- *What were the strategies involved in bringing about change?*
- *If your schools' admissions have mainly remained unchanged, why do you think that is? Were there any attempts to change it?*

3. **Within any medical school, many people – administrators, faculty, students, staff – will have different ways of interpreting "diversity and inclusion." Can you describe some the ways in which diversity and inclusion are <u>discussed</u> at your school?**

Probes:

- *Do faculty and administrators generally agree about the school's priorities with respect to diversity?*
    - *If so, how do you think this agreement has been possible?*
    - *If not, what do you think are the challenges to reaching consensus?*
- *How do you think perspectives on diversity have changed over time at your school?*
- *In your school, how do the conversations differ for discussing racial and ethnic diversity, versus diversity by sexual orientation and gender identity, versus disability, versus other types (as mentioned by participant, e.g. immigration status, religion, political views, etc.)?*
- *In your experience, are there strategies or approaches that are helpful to achieving consensus on*
    - *what diversity means at your school?*
    - *how to prioritize diversity interests versus others?*

4. **Now I'd like to discuss in more detail how your school approaches diversity and inclusion in admissions.**
    a. **What are your school's goals for diversity and inclusion?**
    b. **How does your admissions process address those goals?**

Probes:

- *In your school, who are considered the key stakeholders in diversity and inclusion initiatives?*
    - *To what extent are they included in these decisions?*
- *At your school, who is responsible for:*
    - *Deciding what constitutes "diversity and inclusion"?*
    - *Figuring out how the admissions process will address the school's goals for diversity and inclusion?*

© 2023 Ko M et al. *JAMA Network Open*.

- *If your school uses holistic review, do the criteria involve diversity & inclusion priorities?*
    - *How are the components given relative weight?*
    - *How do you or others decide the relative importance of each component?*
- *How does your school define underrepresented in medicine?*
    - *How was this definition created?*
    - *How often is this definition revised?*
    - *How is this definition used in admissions?*
- *At which stages do you see diversity and inclusion being involved in the admissions process?*
    - *Examples: preparation of diverse pre-medicine students, recruitment of diverse applicant pool, selection of diverse committee members, inclusion of diversity factors at each of the components/stages described in (1), final consideration?*
    - *To the best of your knowledge, how were the decisions made to address diversity and inclusion at these stages?*
- *What types of concerns are brought up by others (faculty/administrators/students/staff) at your school when discussing diversity and inclusion in admissions?*
- *Do members of your committee receive any training or preparation in diversity and inclusion prior to their involvement in admissions?*
    - *What does that training involve?*

5. **All medical schools now have to meet LCME accreditation standards for addressing diversity and inclusion** (*read and provide attached copy of Standard 3 on page 4 if participant is not familiar with the LCME standards*). **Every school has its own set of challenges in meeting these standards, particularly with respect to admissions. Can you describe the challenges at your own school, and how they are being or have been addressed?**

Probes:

- Similar to probes in 1-4, if not already discussed*: How does your school interpret these standards?*
    - *Who is involved in the interpretation?*
    - *Who are the relevant stakeholders, and are they all included?*
    - *Who is involved in developing the school's policies to meet these standards?*
- *In your opinion, how well is your school meeting these standards?*
    - *What makes your own school successful/not successful?*
- *With healthcare changing so rapidly, it can be difficult to balance all the different expectations for medical schools (e.g. changing technology, the push for inter-professional training) how do you and your colleagues (or leadership) decide what to prioritize in admissions?*
- *Researchers in other countries have described how medical school admissions reflect the school's culture. Can you describe in what ways your school's approach to diversity and inclusion reflect the culture of your school?*

Functions and Structure of a Medical School
Standards for Accreditation of Medical Education Programs Leading to the MD Degree Liaison Committee on Medical Education, March 2018, for 2019-2010

3.3 Diversity Pipeline and Partnerships*: A medical school has effective policies and practices in place, and engages in ongoing, systematic, and focused recruitment and retention activities, to achieve mission-appropriate diversity outcomes among its students, faculty, senior administrative staff, and other relevant members of its academic community. These activities include the use of programs and/or partnerships aimed at achieving diversity among qualified applicants for medical school admission and the evaluation of program and partnership outcomes.*

Benefits of diversity*: In a medical education program, the facts that having medical students and faculty members from a variety of socioeconomic backgrounds, racial and ethnic groups, and other life experiences can: 1) enhance the quality and content of interactions and discussions for all students throughout the preclinical and clinical*

© 2023 Ko M et al. *JAMA Network Open*.

curricula; and 2) result in the preparation of a physician workforce that is more culturally aware and competent and better prepared to improve access to healthcare and address current and future health care disparities. (Standard 3)

© 2023 Ko M et al. *JAMA Network Open*.

eTable. Detailed Examples of Strategies Used by Admissions Leaders to Increase Diversity and Inclusion in US Medical Schools

**Theme**	**Strategy**	**Specific actions**
**Change the story**		
	Reframe responsibility of admissions	Define admissions as selection for potential, medical education, and student affairs as responsible for student success
	Incorporate racial justice into institutional mission	Define school mission as specific service to underserved minority populations
		Revise school mission in acknowledgement of racist history of institution and need for repair
	Tailor narratives for messenger and context	‘Data driven’ narratives may be more effective when advocated by those who fulfill traditional academic medicine assumptions of credibility
		Personal experience narratives reinforce credibility of leaders of color when advocating for diversity reforms
**Change the process**		
	Development and implementation of mission-specific admissions criteria	
	Employ rubrics with mission-specific review criteria	Rate applicants for evidence of commitment to underserved communities and understanding of racism and health equity
	Add mission-specific evaluation items	Add questions to supplemental applications and interviews on commitment to, and understanding of, social justice, experiences of racism, and social determinants of health
	Weight mission criteria on par or greater than academic metrics	Present and weigh socioeconomic disadvantage in conjunction with MCAT and GPA at screening
		Accelerate review and presentation of high ‘mission-fit’ applicants to capture earlier in cycle
		Grant mission criteria preferences for waitlist selection
		Tailor scholarship offerings based on mission fit
	Remove ‘hidden’ preferences	Eliminate processes connected to faculty, alumni, or other connections
		Raise legal and publicity concerns
		Require preference requests in writing
		Blind review to names and photos
	Change the weight and use of academic metrics	
	Change benchmarks for academic metrics	Reference metrics to national performance of URiM or socioeconomically disadvantaged applicants
		Use minimum accepted scores, rather than prior cohort or national medians

© 2023 Ko M et al. *JAMA Network Open*.

**Theme**	**Strategy**	**Specific actions**
		Draw lessons from HBCUs, e.g. students with broader range of metrics still demonstrate success
	Change use of academic metrics	Blind/eliminate academic metrics at interview and final review
	Use a continuous quality improvement approach	
	Ongoing review of diversity efforts	Conduct detailed audit of all 'standard' procedures to identify processes that disadvantage URiM applicants
		Leverage admissions data systems to track diversity of applicants at each stage in the cycle
		Conduct reviews beginning, mid, and end of cycle
	Provide feedback to all participants in the process	Flag interviewers for feedback and training if evidence of bias emerges in reports
		Review individual committee member performance and provide personalized recommendations
	Change outcome measures used to gauge success	Employ social justice measures for evaluation of matriculating and graduating students
		URiM representation measured at every stage of cycle
**Change the people**		
	Recruitment	Arrange visits, communication, and partnerships with minority-serving institutions
		Create dedicated team of URiM staff with resources dedicated to recruitment
		Provide frequent and open lines of communication with URiM applicants, invite to revisit, and provide feedback on applications and performance
		Engage URiM students and alumni in recruitment: contacting, mentoring, and hosting applicants
		Provide scholarships for URiM students
	Pathway programs	Create pathway partnerships with minority-serving institutions and guarantee preferences, automatic interviews and/or admissions
		Create programs with local communities, including K-12 and community colleges
	Members of the admissions committee	Recruit URiM faculty, students and community members and provide compensation
		Set expectations with authority to remove members and/or reconstitute committee; institute term limits

© 2023 Ko M et al. *JAMA Network Open*.

		Interview prospective committee members for alignment and understanding of institutional mission
**Theme**	**Strategy**	**Specific actions**
		Foster 'safe space' by establishing standard procedures for managing disagreement, allow for anonymous voting
		Create a separate committee for evaluation of disadvantaged applicants, consisting of members with relevant personal experience
	Educate resident and faculty units on diversity efforts	Admissions members educate residency program leaders on holistic review
		Invite senior departmental leaders to serve on admissions committee
**Change the organization**		
	Integration of other medical school units with shared responsibility to diversity	Leadership holds positions in other units, e.g. student affairs, curriculum, faculty development, graduate medical education
		Regular meetings with different units on shared strategy
		Increase diversity of staffing across all units
	Elevate admissions leadership	Institute direct reporting to higher levels of school leadership rather than education, curriculum, or other levels
	Increase student representation and engagement across units	URiM students serve on admissions committee, in student affairs, and medical education
		Create pathways by which student-led initiatives can inform or become part of school programming/policy
	Provide student support services specific to URiM student needs	Create tailored support: coaching programs, small group learning communities, formal mentorship with residents and faculty
		Offer mental health services providers who understand social structural issues of URiM students
		Identify and address unmet financial needs

© 2023 Ko M et al. *JAMA Network Open*.

	Require health equity training in curriculum	Include content on diversity in medicine, social justice, social determinants of health, health of URiM communities, racism
**Theme**	**Strategy**	**Specific actions**
	Commit resources to Offices of Diversity, Equity and Inclusion	Provide full salary support for ODEI leadership
		Provide staff and funding for ODEI
		Ensure ODEI leadership has necessary authority to implement, not only recommend, change
		Coordinate recruitment, committee member training, admissions metrics with ODEI

© 2023 Ko M et al. *JAMA Network Open*.
