# Supplementary material for: US Medical School Admissions Leaders’ Experiences With Barriers to and Advancements in Diversity, Equity, and Inclusion
Source: JAMA Netw Open. 2023 Feb 24;6(2):e2254928. doi: 10.1001/jamanetworkopen.2022.54928 (PMC9958522; doi:10.1001/jamanetworkopen.2022.54928)
Supplement: Supplement 2. — Data Sharing Statement [file jamanetwopen-e2254928-s002.pdf]

Data Sharing Statement

Ko. US Medical School Admissions Leaders' Experiences With Barriers to and Advancements in Diversity, Equity, and Inclusion. *JAMA Netw Open*. Published February 24, 2023. doi:10.1001/jamanetworkopen.2022.54928

Data
Data available: No

Additional Information
Explanation for why data not available: Data collected for this study were approved by IRB as confidential: identification of participants and institutions poses employment and discrimination risk to the participants.
